# Performance of point-of-care CD4 testing technologies in resource-constrained settings: a systematic review and meta-analysis

**DOI:** 10.1186/s12879-016-1931-2

**Published:** 2016-10-21

**Authors:** Minh D. Pham, Paul A. Agius, Lorena Romero, Peter McGlynn, David Anderson, Suzanne M. Crowe, Stanley Luchters

**Affiliations:** 1 Burnet Institute, 85 Commercial Road, Melbourne, VIC 3004 Australia; 2 Department of Epidemiology and Preventive Medicine, Faculty of Medicine Nursing and Health Science, Monash University, Melbourne, Australia; 3 The Alfred Hospital, The Ian Potter Library, Melbourne, VIC Australia; 4 Department of Immunology, Faculty of Medicine Nursing and Health Science, Monash University, Melbourne, Australia; 5 Department of Infectious Diseases, The Alfred Hospital and Monash University, Melbourne, Australia; 6 Department of Obstetrics and Gynecology, International Centre for Reproductive Health, Faculty of Medicine and Health Sciences, Ghent University, Ghent, Belgium

**Keywords:** CD4, Point of care testing, Diagnostic accuracy, Systematic review, Meta-analysis

## Abstract

**Background:**

Point-of-care (POC) CD4 testing increases patient accessibility to assessment of antiretroviral therapy eligibility. This review evaluates field performance in low and middle-income countries (LMICs) of currently available POC CD4 technologies.

**Methods:**

Eight electronic databases were searched for field studies published between January 2005 and January 2015 of six POC CD4 platforms: PointCare NOW™, Alere Pima™ CD4, Daktari™ CD4 Counter, CyFlow® CD4 miniPOC, BD FACSPresto™, and MyT4™ CD4. Due to limited data availability, meta-analysis was conducted only for diagnostic performance of Pima at a threshold of 350 cells/μl, applying a bivariate multi-level random-effects modelling approach. A covariate extended model was also explored to test for difference in diagnostic performance between capillary and venous blood.

**Results:**

Twenty seven studies were included. Published field study results were found for three of the six POC CD4 tests, 24 of which used Pima. For Pima, test failure rates varied from 2 to 23 % across study settings. Pooled sensitivity and specificity were 0.92 (95 % CI = 0.88–0.95) and 0.87 (95 % CI = 0.85–0.88) respectively. Diagnostic performance by blood sample type (venous vs. capillary) revealed non-significant differences in sensitivity (0.94 vs 0.89) and specificity (0.86 vs 0.87), respectively in the extended model (Wald χ^2^(2) = 4.77, *p* = 0.09).

**Conclusions:**

POC CD4 testing can provides reliable results for making treatment decision under field conditions in low-resource settings. The Pima test shows a good diagnostic performance at CD4 cut-off of 350 cells/μl. More data are required to evaluate performance of POC CD4 testing using venous versus capillary blood in LMICs which might otherwise influence clinical practice.

**Electronic supplementary material:**

The online version of this article (doi:10.1186/s12879-016-1931-2) contains supplementary material, which is available to authorized users.

## Background

Increased testing of those at risk of HIV infection and improving access to antiretroviral therapy (ART) particularly in the most HIV-affected regions is a recognized global strategy to end the AIDS epidemic. However, only 15.0 million of an estimated of 36.9 million (40.6 %) HIV-infected individuals have access to ART [1]. Whilst new evidence endorses treatment of all HIV-infected individuals [2, 3] and the World Health Organization (WHO) guideline supports treatment of all individuals regardless of CD4 count [4], the current practical goal, one that is likely to apply in most resource limited countries into the foreseeable future, is to achieve ART coverage of all HIV-infected adults with low CD4 cell count (<350 cells/μL) before expanding ART scale-up to people with higher CD4 cell counts. Furthermore, for patients who are presenting late to care, a CD4 count is required as a baseline measurement to identify the need for screening and prophylaxis for major opportunistic infections which are often associated with low CD4 count and increased risk of mortality. For treatment monitoring, CD4 count is important to assess CD4-related risk of toxicity to Nevirapine and, CD4 testing remains an important method to monitor patients who are on treatment in settings where access to viral load monitoring is still limited [5]. Thus, CD4 monitoring remains an essential and practical component of HIV care in the near future [6].

It has been known that barriers resulting in substantial losses to the continuum of HIV care include poor access to CD4 testing, particularly in disadvantaged and remote areas where laboratory-based CD4 testing by flow cytometry is not available [7, 8]. Point-of-care (POC) testing is an effective strategy to overcome this challenge. Findings from a number of field studies show that POC CD4 testing can have a positive impact on the HIV continuum of care [9–15]. The use of POC CD4 in lower and middle income countries (LMICs) where resources for HIV care are most limited, is expected to produce greatest clinical and economic impacts from both patient and health system perspectives [16, 17]. With a number of POC CD4 technologies available or in the pipe-line [18], there is a need for consolidated evidence on the performance of different POC CD4 tests, particularly in LMICs, to inform decision-making related to selection of an appropriate test in field settings. A systematic review and a meta-analysis of test performance of CD4 count technologies showed that POC CD4 test is suitable for ART eligibility assessment at CD4 thresholds of 350 and 500 cells/μL [19, 20]. However, these studies pooled data from both laboratory and field evaluations in low and high income countries. As there is evidence to suggest that performance of the test varies significantly across evaluation settings (laboratory vs clinic) in different countries [21, 22], the question remains whether these study findings are transferable, specifically to non-laboratory environments in LMICs. We, therefore, conducted a systematic review to assess the performance, acceptability and feasibility in non-laboratory field settings of currently available or prototype commercial POC CD4 tests in LMICs. Here, we report on “field performance” of different POC CD4 tests; findings on “acceptability, feasibility” will be reported elsewhere.

## Methods

This systematic review was conducted according to a protocol developed using the preferred reporting items for systematic reviews and meta-analyses (PRISMA) statement [23].

### Literature search strategy

The search strategy was designed to identify any studies describing POC CD4 tests. After an initial search for articles in Medline and Embase, an assessment of text words within the title and abstract and of the index terms used to describe these articles was conducted. A subsequent full search using clearly established search terms (see Additional file 1: Annex 1) was undertaken across included databases, and adapted as appropriate to the specifications for the respective databases: Medline, Embase, CENTRAL, Cinahl, PsycINFO, Biological Abstracts, and Scopus. Web of Science and conference databases were also searched to identify relevant studies. Reference lists of all identified reports and articles were searched for additional studies. Moreover, searches were conducted in grey literature resources such as conference websites (NLM Gateway, the British Library Conference and International AIDS Society and Conference on Retroviruses and Opportunistic Infections) and clinical trials websites. Hand-searching and reference checking of citations and reference lists was undertaken. Authors of relevant studies were contacted if insufficient data were published. Government reports, letters to editors, commentaries, editorials, non-peer reviewed articles and review articles were excluded. Studies conducted and funded by the manufacturer, if stated, were also excluded.

### Study selection

**Fig. 1:**
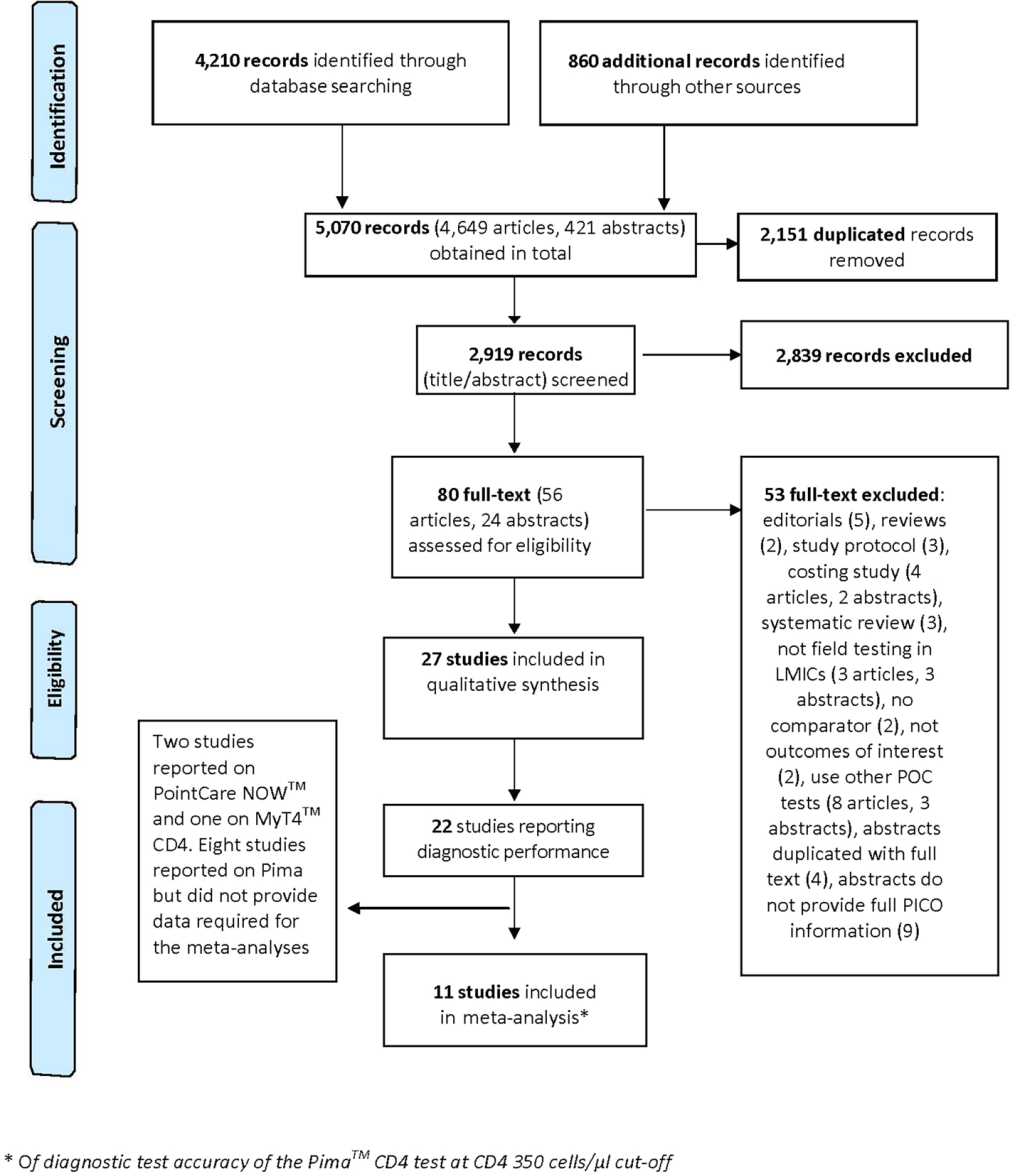
Selection process of included studies

Inclusion criteria were that studies needed to be published between January 2005 and January 2015, written in English language and conducted in LMICs (http://data.worldbank.org/about/country-and-lending-groups). Studies conducted in both LMICs and non-LMICs were included if data from LMICs were presented separately. Further eligibility criteria were defined using PICO (participants, interventions, comparisons, outcomes) format [24]. Participants (P) included HIV positive, HIV negative and unknown HIV status persons aged ≥ 12 months. For intervention (I), any of the following six commercially available POC CD4 testing platforms listed in the UNITAID “2014 HIV/AIDS Diagnosis Technology Landscape” report [18] were included: (1) PointCare NOW™ (PointCare Technology Inc, Marlborough, MA, USA); (2) Pima™ CD4 (Alere Inc, Waltham, MA, USA); (3) Daktari™ CD4 Counter (Daktari Diagnostics Inc, Cambridge MA, USA); (4) CyFlow® CD4 miniPOC (Partec, Munich, Germany); (5) BD FACSPresto™ (BD Biosciences, San Jose, CA, USA); and (6) MyT4™ CD4 Test (Zyomyx Inc, Fremont, CA, USA). Results of POC CD4 test needed to be compared (C) to reference laboratory-based assays with outcomes (O) containing diagnostic performance of POC CD4 test in field settings. All retrieved articles were checked for duplication; conference abstracts were excluded if duplicated with full-text articles. Titles, abstracts and summaries of identified records were screened for relevance. Retained records meeting the inclusion criteria were then examined in full text (Fig. 1).

### Data extraction and data analysis

An electronic data extraction form was developed, pre-tested and finalized by consensus among authors. Data extraction was conducted by one reviewer and verified by the second using the data extraction form with 20 % duplicate extraction. Quality of included studies was assessed using EPHPP tool for quantitative studies [25] and QUADAS tool for diagnostic accuracy studies [26].

Key measures used to evaluate performance of POC CD4 tests were: (1) failure rates defined as the percentage of the total number of tests performed with an invalid result such that tests did not provide results and/or results could not be read. (2) Sensitivity and specificity of index POC CD4 test at certain CD4 thresholds as compared to the predicate method. (3) Misclassification of index POC CD4 at certain CD4 thresholds. Misclassification is defined as the percentage of total HIV-infected individuals/blood samples tested with disagreement in results between POC CD4 and predicate method for the purpose of identifying ART eligibility at pre-specified CD4 threshold values of 200, 350 and 500 cells/μl; and (4) Difference in mean (bias) of CD4 counts and limit of agreement between index POC CD4 and predicate method. Bias included absolute and relative bias defined as the mean absolute and/or relative difference in CD4 count between the index POC CD4 and the predicate test. Limit of agreement was calculated as the mean difference ± 1.96 standard deviation of the difference.

A meta-analysis of diagnostic test accuracy (DTA) was conducted of the Alere Pima™ CD4 (Pima) test through application of a bivariate multi-level random effects modeling approach [27], recommended for meta-analysis for Cochrane DTA reviews. The bivariate random-effects model accounts for both the correlation between study sensitivity and specificity estimates, and also unobserved between-study heterogeneity in test performance through specification of a multi-level bivariate normal regression approach. The bivariate model estimates sensitivity and specificity by modeling random-effects across two levels; level 1 representing the within-study variability between sensitivity and specificity and level 2 representing heterogeneity in diagnostic performance of the index test across studies. Using the multi-level bivariate model; sensitivity, specificity, positive and negative likelihood ratios (±LR) of the Pima test were estimated. In order to test for any difference in diagnostic performance across venous and capillary sample methods, the multi-level bivariate random-effects model was extended to include a covariate for blood sample type. Post-estimation Wald tests were used to test the joint (i.e. for sensitivity and specificity simultaneously) effect of blood sample type on diagnostic accuracy. In all multi-level analyses of pooled Pima test diagnostic performance, Huber/White variance estimation was used to provide appropriate standard errors in instances where multiple sets of diagnostic data were taken from a single study [28]. Diagnostic statistics (Cook’s distance [using a 5 parameter model based cutoff >2] and standardized residual plots) from multi-level bivariate random-effect models were examined to assess pooled sensitivity and specificity estimates for outlier bias. Using Stata version 13.1 (Stata Corporation, TX, USA), both the user-written Stata program *Midas* [29] and author-written code using *GLLAMM* (Generalized Latent and Linear Mixed Modeling) [30] were used to provide key statistical output for pooled multi-level bivariate random-effect model analyses of the diagnostic performance of the Pima test. The user-written Stata program *metandiplot* [31] was used to plot the hierarchical summary receiver operating characteristic (HSROC) curve [32] from observed study sensitivity and specificity estimates. Statistical significance was assessed at 5 % in all analyses.

## Results

### Study characteristics

The initial search, after removing duplicates, yielded 2,919 records, among which 27 studies met all of the inclusion criteria and comprised 24 full-text articles and three conference abstracts [33–35].

Twenty four studies (24) used Pima, two studies used PointCare NOW, one used MyT4 as the index POC CD4 test. Overall, the quality of included studies was considered between moderate and strong. The QUADAS scores ranged from 7 to 12 (of a maximum score of 14) with 63 % (12/19) of studies scoring between 10 and 12. Among 22 studies reporting performance of POC CD4 test (Table 1), 19 (86 %) were conducted in sub-Saharan countries and three others were in India [36], Brazil [37] and PNG [38]. CD4 predicate testing technologies used as reference include FACSCalibur, FACSCount, Panleucogating (PLG) flow cytometry, Partec CyFlow and GUAVA. Only one study performed duplicate testing [39], and three studies performed precision testing of the reference method on a subset of whole blood samples (3 – 15 blood specimens) [40–42]. Among 19 studies reporting diagnostic performance of Pima, only 11 studies provided data required for and then subsequently included in the meta-analyses.

**Table 1:** Characteristics and Quality assessment of studies included in the review

First author, year	Study population/Study setting	Study design/Sample size	Sample	Intervention	Comparison	Study quality^a^
Rathunde, 2014 [37]	Adult HIV patients at specialized ambulatory facilities in academic tertiary care hospital in Curitiba, Brazil	Cross-sectional (*N* = 107)	Venous	Alere Pima™ CD4 (Pima)	FACSCalibur	9
Galiwango, 2014 [49]	HIV infected patients (pre and experienced ART persons) at field clinics in Rakai, Uganda	Cross-sectional (*N* = 903)	Venous	Pima	FACSCalibur	10
van Rooyen, 2013 [15]	Known HIV-positive individuals older than 18 years in KwaZulu-Natal, South Africa (SA)	Prospective cohort (*N* = 671)	Capillary (Pima); venous (FACSCalibur)	Pima	FACSCalibur	Moderate
Myer, 2013 [45]	HIV infected pregnant women at a single large antenatal clinic in Cape Town, SA	Cross-sectional (*N* = 546)	Venous	Pima	Beckman-Coulter PLG technology	8
Mnyani, 2012 [51]	HIV infected pregnant women at first ANC visit to Chiawelo clinic in Johannesburg, SA	Cross-sectional (*N* = 305)	Capillary (Pima); venous (Ref. tests)	Pima	Beckman Coulter Flow Cytometer	10
Mwau, 2013 [53]	Patients attending 9 health facilities (for HIV treatment and care) offering CD4 count in Kenya	Cross-sectional (*N* = 1539)	Venous (Pima & reference methods); capillary (Pima)	Pima	FACSCount, Partec Cy-flow, GUAVA and FACSCalibur	8
Glencross, 2012 [22]	Adult HIV patients attending (1) Hospital based antenatal HCT clinic in Johannesburg-phase II (2) Two Primary health care HCT clinic in Limpopo province-phase IIIA; and (3) Inner-city primary health care clinic in Johannesburg, South Africa-phase IIIB	Cross-sectional (*N* = 91; *N* = 96; *N* = 139)	Venous and capillary	Pima	Simplified single platform (SP) PLG CD4	12
Thakar, 2012 [36]	HIV positive patients aged 18–60 attending 21 ART centers in different parts of India	Cross-sectional (*N* = 1790)	Venous and capillary	Pima	FACSCalibur; FACSCount; Partec CyFlow	12
Manabe, 2012 [46]	HIV infected patients at Adult Infectious Diseases Institute Clinic in Mulago Hospital, Kampala, Uganda	Cross-sectional (*N* = 206)	Venous and capillary	Pima	FACSCalibur	11
Jani, 2011 [39]	HIV infected individuals attending 2 primary health care ART clinics in Maputo, Mozambique	Cross-sectional (*N* = 697)	Capillary (Pima); venous (Ref. tests)	Pima	FACSCalibur	7
Diaw, 2011 [44]	Patients (adults & children; HIV +/−) presenting for HIV follow-up at three out-patient clinic & one lab in Dakar, Senegal	Cross-sectional (*N* = 300)	Venous (200 patients); capillary (finger/heel-prick) 100 patients	Pima	FACSCount	11
Mtapuri-Zinyowera, 2010 [50]	Newly diagnosed HIV positive patients at a VCT center in Harare, Zimbabwe	Cross-sectional (*N* = 165)	Capillary (Pima); venous (Ref. tests)	Pima	FACSCalibur	11
Wade, 2014 [40]	HIV infected patients presenting for routine CD4 testing at infectious disease clinic in Dar es Salam, Tanzania	Cross-sectional (*N* = 200)	Capillary (Pima); venous (Ref. tests)	Pima	FACSCalibur	11
Wade, 2013 [42]	(Anonymous) HIV infected patients attending normal CD4 test monitoring and HIV-negative donors from blood bank of Regional Hospital and two Healthcare Centers in Ziguinchor, Senegal	Cross-sectional (*N* = 128)	Venous	Pima	FACSCount	11
Gous, 2013 [52]	HIV infected patients > 18 years old visiting comprehensive care management and treatment clinic for ART initiation or monitoring at Tshwane District Hospital in Pretoria, SA	Cross-sectional (*N* = 300)	Capillary (Pima); venous (Ref. tests)	Pima	PLG CD4 FC 500	10
Malagun, 2014 [38]	HIV infected adults >18 year old attending one urban (Heduru HIV clinic at Port Moresby General Hospital, Port Moresby) and 2 rural (Asaro District Health Centre and Kainantu Rural Hospital) clinics in Papua New Guinea	Cross-sectional (*N* = 237)	Venous	Pima	FACSCount	11
Picken, 2014 [43]	HIV positive mother of children with CD4 count < 500 cells/μl and not on ART at Tygerberg hospital, Cape Town, SA	Cross-sectional (*N* = 52)	Capillary (Pima); venous (Ref. tests)	Pima	Beckman Coulter FC 500 MPL®	11
Mwau, 2014 [48]	HIV infected patients ≥ 18 years old at Comprehensive clinics of 2 health care facilities in Busia county of Western province, Kenya	Cross-sectional (N = 276)	Capillary (Pima); venous (Ref. tests)	MyT4 POC CD4	FACSCalibur and FACSCount	11
Gumbo, 2013 [41]	HIV infected adult patients attending Harare Central hospital opportunistic infection clinic, Zimbabwe	Cross-sectional (*N* = 104)	Venous	PointCare NOW	BD FACSCalibur	9
Bergeron, 2012 [47]	HIV infected adult patients; unknown HIV status; and HIV infected children aged 12–59 months attending: (1) the Instituto Nacional de Saude (INS) Maputo, Mozambique; (2) the Institute of Tropical Medicine of Antwerp (Belgium) in Tete, Mozambique; (3) Wits University, Johannesburg, SA	Cross-sectional (*N* = 472)	Venous	PointCare NOW	FACSCalibur and the EPICS- XL	8
Zeh, 2014 [35]	HIV infected patients in Western Kenya	Cross-sectional (*N* = 147)	Venous and capillary	Pima	FACSCalibur	-
Arnet N, 2013 [33]	HIV infected patients from 5 PMTCT and HIV treatment sites in Dar-es-Salaam, Tanzania	Cross-sectional (*N* = 1060)	Venous and capillary	Pima	FACSCalibur	-

*HCT* HIV counseling and testing, *ART* Antiretroviral therapy, *VCT* Voluntary counseling and testing; *PMTCT* Prevention of mother to child transmission

^a^Study quality assessment using *EPHPP tool (strong/moderate/weak), or QUADAS score (out of 14)*

### Performance of POC CD4 tests in field settings

#### Failure rates

For Pima, studies with capillary blood reported a wide range of failure rates from 2 % [43] to 23.3 % [44]. Studies using venous blood in various clinical settings reported failure rates ranging from 4.8 to 15.2 % [40, 44–46]. One study reported a zero “no read” error in laboratory evaluation of Pima with venous blood, however, in field evaluations in different clinical settings failure rate was recorded at a wide range from 6.8 to 20.9 % [22]. For PointCare Now the failure rate varied from 2.9 % in one study [41] to 9.2 % in another [47]. With MyT4 CD4 test a study-wide error rate of 9.6 % was recorded [48].

Detailed performance data of Pima is presented in Table 2.

**Table 2:** Performance of Pima stratified by venous and capillary blood collection and presented by reference test used

Author, year	Performance of Pima on capillary blood				Author, year	Performance of Pima on venous blood			
	Bias/LoA; Sample size (N)	Sensitivity Specificity	Total Misclassification	Failure rate		Bias/LoA Sample size (N)	Sensitivity	Total Misclassification	Failure rate
							Specificity		
*Reference test =* FACSCalibur									
(van Rooyen, Barnabas et al. 2013) [14]	Mean bias: 16 cells/μl (LoA: −1 to 32; *N* = 193)	*Not reported (NR)*	*NR*	*NR*	(Rathunde, Kussen et al. 2014) [32]	*Bias/LoA NR; N* = *107*	At CD4 threshold of 350 cells/μl: Sensitivity 94 % Specificity 93 %	*NR*	*NR*
(Jani, Sitoe et al. 2011) [34]	Accurate absolute counts	*NR*	At CD4 thresholds of 200 cells/μl: 5.2 %	*NR*	(Galiwango, Lubyayi et al. 2014) [45]	Pima significantly underestimate CD4 count particularly at higher CD4 count.	At CD4 threshold of 350 cells/μl: Sensitivity 88.6 % specificity 87.5 %	12.2 %	*NR*
	Bias: −52.8 cell/μl (LoA: −250.9 to 145.2; *N* = 135).								
	Bias was smaller at lower CD4 count (<500: −24.4) than at higher CD4 count (>500: −107.9).		At CD4 thresholds of 350 cells/μl: 17 %			Bias: −34.6 cells/μl (LoA: −219.8 to +150.6; *N* = 903)	At CD4 threshold of 500 cells/μl: Sensitivity 96.1 % specificity 83.0 %	9.5 %	
						*At 350 cut-off*: +5.1 cells/μl (LoA: −126.6 to +136.8) vs −51.0 cells/μl (LoA: −245.4 to +143.4)			
						*At 500 cut-off*:-10.9 cells/μl (LoA: −147.3 to +125.5) vs −66.3 cells/μl (LoA: −286.6 to +154.0)			
(Mtapuri-Zinyowera, Chideme et al. 2010) [46]	Mean bias: 7.6 cells/μl (LoA: −173.8 to +189.0).	At CD4 threshold of 200 cells/μl: Sensitivity: 95.1 % Specificity: 91.6 %	6.7 %	*NR*	(Mwau, Adungo et al. 2013) [49]	Bias: −64.8 cells/μl (LoA: −332.5 to +203.0; *N* = 396)	At CD4 threshold of 350 cells/μl (in those ≥ 5 years old *N* = 389): Sensitivity: 89.7 % Specificity: 87 %	11.9 % (47/396)	*NR*
	Bias was small at both low (<400 cells/μl) and high (>400 cells/μl)	With sub-samples of FACSCalibur results of (100 to 300 cells/μl)	12.8 %				At CD4 threshold of 200 cells/μl (in those ≥ 5 years old *N* = 389): Sensitivity: 86.7 % Specificity: 94.1 %	*NR*	
		At CD4 threshold of 350 cells/μl: Sensitivity: 94.7 % Specificity: 87.5 %	6.7 %						
		With sub-samples of FACSCalibur results of (200 to 500 cells/μl)	14.1 %						
(Thakar, Mahajan et al. 2012) [31]	Relative bias: −9.1 %; LoA: −46 % to 27 %; *N* = 175	*NR*	*NR*	*NR*	(Thakar, Mahajan et al. 2012) [31]	Among patients with CD4 < 350 cells/μl: relative bias: +4 % (*N* = 121)	At CD4 threshold of 350 cells/μl: Sensitivity: 96 %; Specificity: 91 %	*NR*	*NR*
(Manabe, Wang et al. 2012) [41]	Bias: −66.3 cells/μl (LoA: −83.4 to +49.2; *p* < 0.001; *N* = 176)	*NR*	*NR*	17.7 %	(Manabe, Wang et al. 2012) [41]	Bias: −68.5 cells/μl (LoA: −79.6 to −57.4; *p* < 0.001; *N* = 206)	*NR*	*NR*	8.1 %
	Bias was smaller at lower CD4 counts (−10.8 cells/μl; LoA: −27.3 to +5.6; *p* = 0.19 for CD4 range 0–250 cells/μl) and much greater at higher CD4 count (−120.6 cells/μl; LoA: −162.8 to −78.4; *p* < 0.001 for CD4 > 500 cells/μl)					Bias was smaller at lower CD4 counts: +13.6 cells/μl (LoA: 2.52 to 24.7; *p* = 0.02 for CD4 range 0–250 cells/μl) and much greater at higher CD4 counts: −121.7 cells/μl (LoA: −147.9 to −95.4; *p* < 0.001 for CD4 > 500 cells/μl)			
(Wade, Daneau et al. 2014) [35]	Relative bias: −0.9 %; (LoA: −57.3 to +55.6); *N* = 200	At CD4 threshold of 200 cells/μl: Sensitivity: 100 % Specificity: 94 %	4 % (16/410)	4.5 % (9/200)	(Wade, Daneau et al. 2014) [35]	Relative mean bias: −9.4 % (LoA: −54.4 to +35.6)	At CD4 threshold of 200 cells/μl: Sensitivity: 98 % Specificity: 95 %	3 % (14/440)	6.5 % (13/200)
	Sub-samples of CD4 ≤ 200: 5 % (−78 to +89); CD4 200–500: 0 % (−49 to +49); CD4 ≥ 500: −8 % (−49 to +34)	At CD4 threshold of 350 cells/μl: Sensitivity: 87 % Specificity: 90 %	13.4 % (55/410)			Sub-samples of CD4 ≤ 200: 1 % (LoA: −75 to 77); CD4 200–500: −11 % (LoA: −46 to +25); CD4 ≥ 500: −15 % (LoA: −34 to +4)	At CD4 threshold of 350 cells/μl: Sensitivity: 97 % Specificity: 80 %	9 % (40/440)	
		At CD4 threshold of 500 cells/μl: Sensitivity: 97 % Specificity: 82 %	*NR*				At CD4 threshold of 500 cells/μl: Sensitivity: 99 % Specificity: 78 %	NR	
(Zeh, Inzaule et al. 2014) [30]	Bias: −44 cells/μl; *N* = 147	At CD4 threshold of 350 cells/μl: Sensitivity: 86 % Specificity: 99 %	*NR*	*NR*	(Zeh, Inzaule et al. 2014) [30]	Bias: −86 cells/μl; *N* = 147	At CD4 threshold of 350 cells/μl: Sensitivity: 94 % Specificity: 95 %	*NR*	*NR*
(Arnett N 2013) [28]	Bias: 0 (PIMA –Microtube) and −20 cell/μl (PIMA –direct); *N* = 1060	*NR*	*NR*	8.6 % (Micro-tube) and 10.1 % (direct)	(Arnett N 2013) [28]	Bias: −10 cells/μl	*NR*	*NR*	7.7 %
*Reference test =* FACSCount									
(Mwau, Adungo et al. 2013) [49]	Mean bias: +8.6 cells/μl (LoA: −235.4 to 252.7; *N* = 521)	At CD4 threshold of 350 cells/μl: Sensitivity: 79.4 % Specificity: 86.9 %	16.5 % (86/521)	*NR*	(Mwau, Adungo et al. 2013) [49]	Mean bias: +7.8 cells/μl (LoA: −168.9 to 184.4; *N* = 822)	At CD4 threshold of 350 cells/μl (in those ≥ 5 years old *N* = 813): Sensitivity: 79.4 % Specificity: 83.4 %	*NR*	*NR*
							At CD4 of 200 cells/μl (in those ≥ 5 years old *N* = 813): Sensitivity: 83 % Specificity: 98.2 %	*NR*	
					(Thakar, Mahajan et al. 2012) [31]	Among patients with CD4 < 350 cells/μl: Mean relative bias: −5 % (*N* = 206)	At CD4 threshold of 350 cells/μl: Sensitivity: 92 %; Specificity: 91 %	*NR*	
(Diaw, Daneau et al. 2011) [39]	Of 95 HIV (+) patients, Absolute bias: −39 cells/μl (LoA: −258 to +179)	At CD4 threshold of 200 cells/μl: Sensitivity: 91 %	5.3 % (5/95); of finger-prick samples	14 % total; 23 % in one study site	(Diaw, Daneau et al. 2011) [39]	For 100 HIV(+) patients, Absolute bias: −32 cells/μl (LoA: −146 to +84)	At CD4 threshold of 200 cells/μl: Sensitivity: 90 %	4 %	4.8 %
		Specificity: 97 %					Specificity: 98 %		
	Sub-samples of CD4 < 200: bias: +15 cells/μl (LoA: −89 to 118); Sub-samples of CD4 > 500: bias: −112 cells/μl (LoA: −429 to 204)	At CD4 threshold of 350 cells/μl: Sensitivity: 91 %; Specificity: 80 %	*NR*			Sub-samples of CD4 < 200: bias: +9.4 cells/μl (LoA: −76 to 94); Sub-samples of CD4 > 500: bias: −77 cells/μl (LoA: −217 to 63)	At CD4 threshold of 350 cells/μl:	*NR*	
							Sensitivity: 98 %		
							Specificity: 79 %		
						For 99 HIV(−) controls Absolute bias: −125 cells/μl (LoA: −434 to +184 cells/μl for all ranges of CD4			
					(Wade, Diaw et al. 2013) [37]	Bias: −30 cells/μl (LoA: −160 to 101; *N* = 128: 111 HIV+ & 17 HIV-)	At CD4 threshold of 200 cells/μl:	*NR*	*NR*
							Sensitivity 95 %		
							Specificity 96 %		
						Sub-samples of CD4 < 200: Bias: +6.0 cells/μl (LoA: −39 to +51)	At CD4 threshold of 350 cells/μl:		
							Sensitivity 97 %		
							Specificity 90 %		
						Sub-samples of CD4 > 500: Bias: −65 cells/μl (LoA: −224 to +93)	At CD4 threshold of 500 cells/μl:: Sensitivity 99 % Specificity 72 %		
					(Malagun, Nano et al. 2014) [33]	Urban clinic: Bias: −46.4 cells/μl (LoA:-199.8 to 107.0); *N* = 139	At CD4 threshold of 350 cells/μl: Sensitivity: 99.2 %; specificity: 77.1 %	10.7 %	Error rate: 5.1 %
						Rural clinic: Bias: −55.8 cells/μl (LoA: −182.9 to 71.2); *N* = 98			
*Reference test =* Beckman-Coulter flow cytometry using Pan-leucogating (PLG) method									
(Mnyani, McIntyre et al. 2012) [47]	Bias: −20.5 cells/μl (LoA: −175.0 to +133.9; *p* < 0.001; *N* = 296)	At CD4 threshold of 350 cells/μl:	10.8 %; mostly in favor of patient treatment.	*NR*	(Myer, Daskilewicz et al. 2013) [40]	Bias: −22.7 cells/μl (LoA: −174.6 to 129.2); *N* = 546.	At CD4 threshold of 350 cells/μl:	10 %	61/546 samples required 83 additional test; 4 returned no result due to repeated machine errors
	No significant variability in the level of agreement related to age and gestational age	Sensitivity: 93 % (95 % CI 87–96), Specificity: 86 % (95 % CI 80–91)				Bias increased with increasing gestational age	Sensitivity: 92 %		
							Specificity: 89 %;		
							Sensitivity & specificity did not vary significantly across gestational age		
(Glencross, Coetzee et al. 2012) [42]	Phase II (Hospital ANC clinic: Bias: −37.9 cells/μl (LoA: −389.1 to 309.8; *N* = 77	*NR*	*NR*	*NR*	(Glencross, Coetzee et al. 2012) [42]	Phase II (Hospital ANC clinic: Bias: −19.6 cells/μl (LoA: −149.1 to 110.0; *N* = 91)	*NR*	*NR*	10.4 % (5/48) & 20.9 % (9/43) for 2 devices
	Phase IIIA Rural/poor resourced clinic: Not applicable *(NA)*	*NA*	*NA*	*NA*		Substantial, clinically significant difference to predicate: Bias +105.7 cells/μl (LoA −336.1 to 547.5; *N* = 96)		Among 32 patients with CD4 < 350: 10 patients (31.2 %) would have missed ART (upward misclassification)	6.8 % (7/103)
						Larger bias and wider LoA for samples with CD4 < 350: +131.4 cells/μl (LoA: −275.8 to +538.6; *N* = 32) as compared to samples with CD4 < 500: +102.3 cells/μl (LoA: −289 to 493.6; *N* = 52) = > increasing error at CD4 range of less than 350 cells/μl			
	Phase IIIB well resourced clinic: *NA*	*NA*	*NA*	*NA*		Results showed considerably less bias and tighter LoA variance, as compared to phase IIIA, irrespective of lancet used: lancet 1 (Sarstedt) bias: +8.9 cells/μl (LoA: −211.1 to 229; *N* = 87); lancet 2 (Caralet Blue) bias: −11.2 cells/μl (LoA: −147 to 124; *N* = 52)			9 % (14/153)
(Gous, Scott et al. 2013) [48]	Phase I: Multiple POC testing from multiple finger-sticks: mean bias was −32 cells/μl (*N* = 98) PIMA overestimate at low CD4 count (<350) and underestimate at high CD4 count (>500 cells/μl)	At CD4 threshold of 350 cells/μl: Sensitivity 86.4 %, Specificity 88.5 %	12.4 %	16.3 %		*NA*	*NA*	*NA*	*NA*
	Phase II: Multiple POC testing from single finger-stick: Mean bias - 30 cells/μl (*N* = 73)	At CD4 threshold of 350 cells/μl: Sensitivity 97.5 %, Specificity 95 %	4.1 %;	19.2 %		*NA*	*NA*	*NA*	*NA*
(Picken, Williams et al. 2014) [38]	Bias: 23.8 cells/μl (LoA: −166.1 to 213.8; *N* = 50	At CD4 threshold of 350 cells/μl: Sensitivity: 88.9 %, specificity: 90.6 %	10 %	1.9 %					
*Reference test =* Partec Cyflow									
(Mwau, Adungo et al. 2013) [49]	Mean bias: −10.0 cells/μl (LoA: −261.4 to 241.4; *N* = 162)	*NR*	*NR*	*NR*	(Mwau, Adungo et al. 2013) [49]	Mean bias: −24.2 cells/μl (LoA: −277.6 to +229.3; *N* = 407)	*NR*	*NR*	*NR*
					(Thakar, Mahajan et al. 2012) [31]	Among patients with CD4 < 350 cells/μl: mean relative bias +8 % (*N* = 550)	At CD4 350 threshold: Sensitivity: 91 %; Specificity: 96 %	*NR*	*NR*
*Reference test =* GUAVA									
(Mwau, Adungo et al. 2013) [49]	Mean bias: +23.9 cells/μl (LoA −329.6 to 281.9; *N* = 176)	*NR*	*NR*	*NR*	(Mwau, Adungo et al. 2013) [49]	Mean bias: −0.3 cells/μl (LoA: −315.0 to 315.6; *N* = 191)	*NR*	*NR*	*NR*

*LoA* limit of agreement, *POC* point-of-care, *ANC* antenatal care

#### Misclassification, sensitivity and specificity

When CD4 count testing was conducted using a venous blood specimen Pima showed lower misclassification and higher probabilities of correctly identifying patients eligible for ART across studies and different reference methods. At a CD4 threshold of 350 cells/μl, the total misclassification probability of Pima test using venous blood was 4.0–12.2 % [44, 49] versus 6.7–17 % for capillary blood [39, 50]. Pima point estimates for sensitivity and specificity ranged from 89–99 % and 77–93 % for venous blood; and 79–98 % and 80–99 % for capillary, respectively. For PointCare Now, one study [47] reported site-specific sensitivity ranging from 38 to 63 %, resulting in misclassification of 50 % of patients tested as ineligible for ART; another study [41] reported a lower misclassification of 6 % of patients as ineligible for treatment. For MyT4 CD4, the sensitivity and specificity of the test were 88 and 84 % when compared to FACSCalibur and 95 and 88 % as compared to FACSCount [48].

##### Meta-analysis of diagnostic accuracy of the Alere Pima™ CD4 in field testing

We aimed to conduct categorical data analysis of diagnostic accuracy of Pima at CD4 350 and 500 cells/μl cut-offs. However, only three included studies [40, 42, 49] reported data required for meta-analysis at the 500 cut-off. Thus only analysis at a CD4 threshold of 350 cells/μl was performed. Required data including number of true positive, false positive, false negative and true negative cases were reported in the literature from 9 studies [38, 40, 42, 43, 45, 49–52]. Data from two studies [37, 53] were received from the authors following email contact, yielding a final dataset comprising 11 studies for the meta-analysis. Among these, two studies [40, 53] reported Pima test results for both venous and capillary blood, and these data were treated in meta-analyses as independent study results but with model standard errors corrected for the lack of independence in observations. Five studies [37, 38, 42, 45, 49] reported the results with venous and four studies [43, 50–52] with capillary blood only.

Examination of post-estimation of diagnostic statistics after preliminary meta-analyses provided some evidence of model outlier bias, with two studies [53] (capillary) and [38] (venous) showing model discrepant test sensitivity and specificity (Cook’s distances: 2.12 and 3.65 respectively). However, diagnostic test data from these studies were included in the pooled meta-analysis after sensitivity analysis, with outlying cases excluded, indicated no marked difference in pooled estimates (included versus excluded sensitivity and specificity: 92 vs. 92 % and 87 vs. 88 %, respectively).

**Fig. 2:**
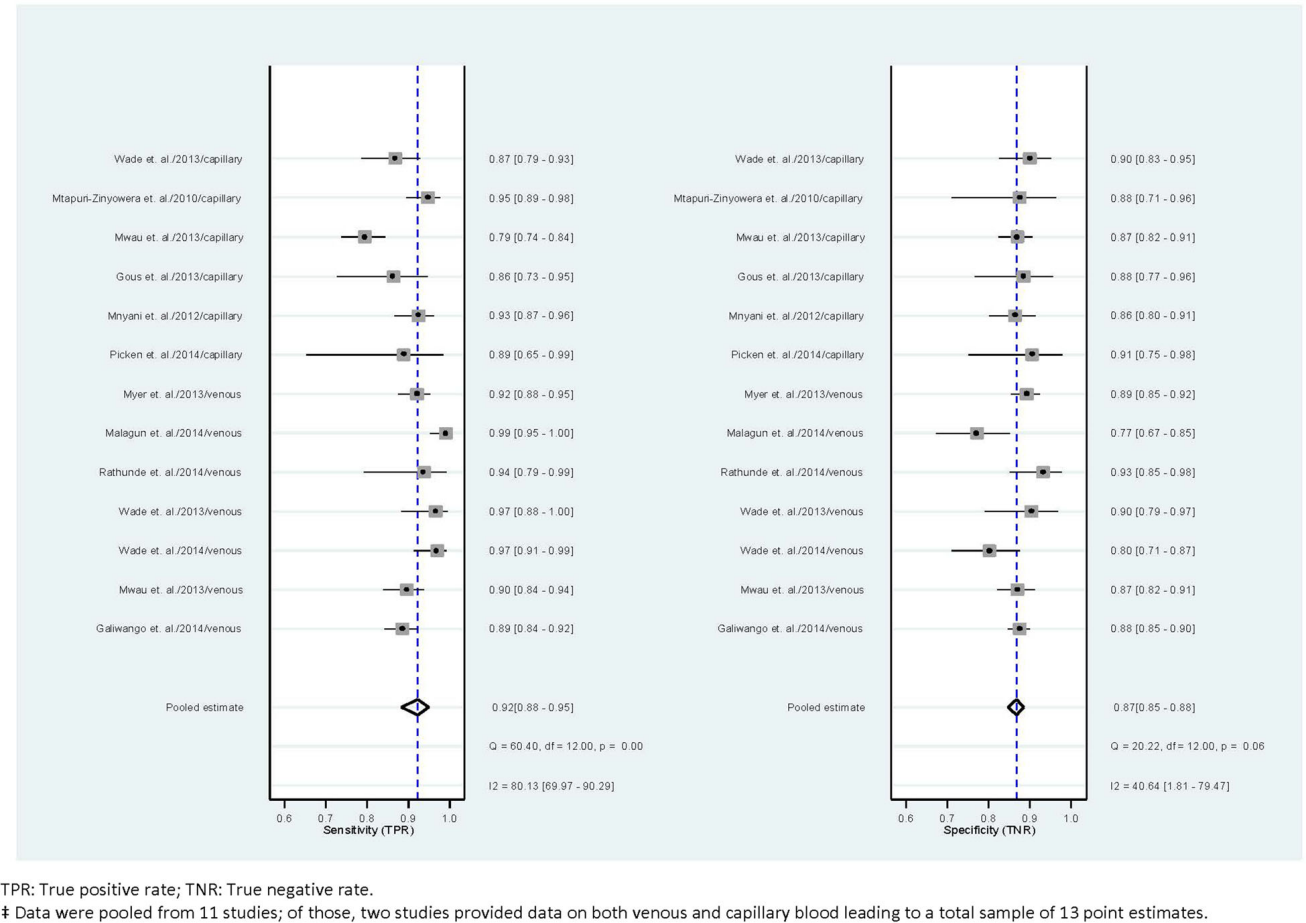
Point estimates^‡^ of diagnostics performance of Pima in field settings at CD4 350 cells/μl cut-off

**Fig. 3:**
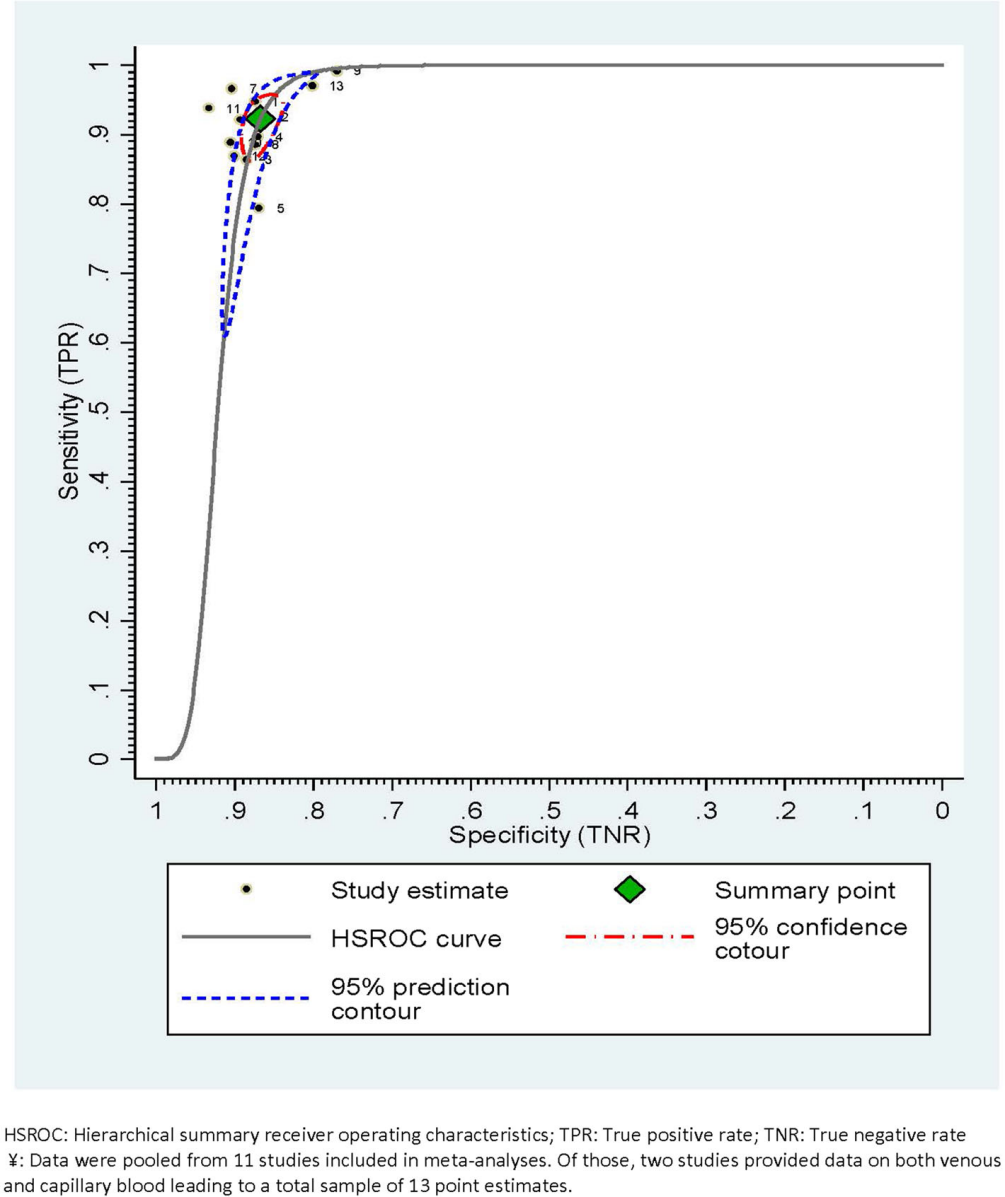
HSROC curve from multi-level bivariate random effects model estimation of diagnostic performance of Pima at CD4 350 cells/μ cut-off^¥^: plots observed sensitivity and specificity, diagnostic summary point, 95 % confidence and prediction contours.

Diagnostic accuracy of the test in field settings was relatively high, with pooled sensitivity and specificity estimated at 92 % (95 % CI = 88–95 %) and 87 % (95 % CI = 85–88 %) respectively (Fig. 2). Further, pooled positive and negative likelihood ratios were also at levels indicating relatively strong diagnostic performance of Pima (+LR = 7.0, 95 % CI = 6.1–7.9; −LR = 0.09, 95 % CI = 0.06–0.13). Figure 3 shows observed sensitivity and specificity plotted for each included study with the HSROC curve, the pooled estimate and 95 % confidence and prediction contours.

Bivariate random-effect hierarchical models estimating pooled diagnostic performance with a covariate for blood sample type showed some potential difference in summary sensitivity and specificity by blood sample type. Using venous samples, pooled sensitivity was 94 % (95 % CI = 89–97 %) and pooled specificity 86 % (95 % CI = 82–89 %), while using capillary blood pooled sensitivity was 89 % (95 % CI = 83–93 %) and specificity 87 % (95 % CI = 86–89 %). However, a post-estimation test of the joint effect of blood sample type on the sensitivity and specificity of Pima showed that these differences in diagnostic accuracy did not reach statistical significance (Wald χ^2^(2) = 4.77, *p* = 0.09).

#### Bias and limit of agreement (LoA)

Overall, Pima showed a better performance with venous compared to capillary blood samples with a smaller range of bias and tighter LoA across studies with different predicate technologies. Studies reported absolute bias of Pima at CD4 > 500 cells/μl ranging from −66.3 cells/μl (LoA: −286.6, +154.0) for venous [49] to −120.6 cells/μl (LoA: −162.8, −78.4) for capillary blood [46]. At lower CD4 ranges, bias were reported at +15 cells/μl (LoA: −89 to +118) for CD4 < 200 cell/μl using capillary blood [44]; and +5.1 cells/μl (LoA: −126.6, +136.8) for CD4 < 350 cell/μl using venous sample [49]. These data suggest that Pima overestimates the CD4 count at lower CD4 ranges and underestimates the CD4 count at higher ranges; the bias was also increased at higher CD4 counts.

## Discussion

Findings of this review suggest that POC CD4 testing can provide reliable results for making treatment decisions among HIV patients in LMICs. This review highlights the need for published data regarding the field evaluation of available POC CD4 tests, particularly in low-resource settings where these novel technologies are already demonstrating significant impact on the continuum of care for HIV-positive persons. Among six current or prospective commercially available POC CD4 technologies, only three have published studies that meet inclusion criteria and most of these used Pima as the index test. Among 19 studies reporting Pima performance data, 11 studies provided data required for meta-analysis of diagnostic test accuracy.

Findings on Pima performance from two studies using both venous and capillary blood showed that CD4 counts on venous blood samples produced more accurate results than capillary blood, with lower failure/error reading rate, the authors suggest that variation in test results was likely due to quality of capillary sampling [22, 44]. However, other evidence supports the use of either venous or capillary specimens [35, 40]. Though not statistically significant, our meta-analysis shows that there is a trend towards a better performance of the test with venous blood, with a sensitivity of 0.94 for venous and 0.89 for capillary blood (*p* = 0.09) in identifying HIV-positive person eligible for ART at a cut-off of 350 cells/μL. If this is a true difference in performance of the test, the use of venous blood when using Pima for ART eligibility assessment would be preferable as it could reduce false negative test results which represent patient’s missed opportunities for timely treatment initiation.

An observed wide range of failure rate of the Pima technology across studies is another attribute that needs further attention. Apart from technical and operational characteristics of the test, evidence from field studies suggests that performance of the test operator influences the accuracy of diagnostic test in the field [11, 22]. Therefore, the quality of training on POC testing for test operators and their supervisors becomes critically important to ensure effectiveness and efficiency of the technology in field settings. Of note, few of the included studies mentioned the effect of staff training on performance of POC CD4 test and none described details of the training program.

Bias in assessing the diagnostic accuracy of any new test could arise from faulty results of the reference test itself as there is no gold standard for CD4 testing although single platform flow cytometry has assumed that position. Thus evidence of participation and successful performance in external quality assurance (EQA) programs and performing duplicate tests on a sample using the predicate test are worthy recommendations in order to ensure the highest accuracy of predicate results. In this review only half of the included studies described EQA participation for the reference test and only one study conducted duplicate testing.

In order to better inform the decision making process on selection and adoption of POC CD4 testing in LMICs, further studies on currently and newly available POC CD4 technologies in various level settings and different geographic regions are needed. It is recommended that the quality of studies as well as quality of study reporting should be improved by following established standards [26, 54] and the focus of these future studies should not only be on test diagnostic accuracy but also on implementation aspects of the test, aiming at providing practical evidence to inform effective implementation strategies of POC CD4 testing.

There are two published systematic reviews and meta-analyses of the performance of POC CD4 tests, one by Scott et al. [19] and one by Peeling et al. [20]. In comparison, our review included 22 peer reviewed publications, providing a large increase in analysis of published work on POC CD4 technologies compared to the other reviews. Importantly, our study differed from the previously published reviews in that *only* studies conducted in field settings were included and thereby specially assessed field performance of the Pima. We demonstrated that misclassification by Pima, particularly with the use of capillary blood samples under field conditions, can be higher than that under a laboratory environment; thus our data vary with the earlier study where the reported probability of Pima misclassification was less than 10 % [20]. A significant methodological strength of our meta-analysis is the direct estimation of the joint effect of blood sample type on Pima sensitivity and specificity simultaneously, using a bivariate multi-level random effect model with pooled study data. This is a significant improvement in statistical robustness compared to the simple comparison of 95 % CI estimates for sensitivity and specificity applied in the other meta-analysis [19]. Encouragingly, our results, in line with findings from other reviews, confirm that POC CD4 tests also perform well if assessed specifically in field settings. Pima therefore has the potential for further deployment for ART eligibility assessment and treatment monitoring, especially in areas where laboratory-based CD4 testing is not available or difficult to access.

This review has some limitations which may affect the generalization of the findings. First, we included only published, peer-reviewed journal articles in English and this inclusion may overlook data from studies published in other languages or unpublished data from evaluations/studies conducted by government agencies, reference facilities or similar institutions. The inclusion of conference abstracts has it strengths in limiting publication bias; however, confidence in these findings is limited as the quality of these studies has not been assessed via formal peer review. Second, only three of the six POC CD4 technologies found in this review were published with field study data and one technology (Pima) featured most prominently in the included studies. This presents challenges in terms of generalizing many of the findings of the review to “all” POC CD4 tests as it may be subjected to reporting bias. Third, interpretation of findings from meta-analyses of this review should be contextualized in terms of the limited diagnostic test data available from published studies. This limitation cannot be overcome until more data from field studies of different POC CD4 technologies, including the Pima, are available.

## Conclusions

Findings of this review suggest that field studies of POC CD4 tests currently available on the market and those eagerly anticipated, conducted in LMICs where they are needed the most, remain much in need. The Pima™ CD4 showed acceptable diagnostic test accuracy using either venous or capillary blood. Existing evidence indicates that POC CD4 testing, can provide reliable results under field conditions and could play an important role in HIV continuum of care. This remains true, despite the changing landscape with respect to guidelines for ART initiation. Whilst evidence supports increasingly earlier commencement of treatment at an individual and community level, the financial reality is that in many parts of the world priority for ART initiation must still continue to be given to those with evidence of declining immune function. Further evidence is needed to ensure that efficacy is acceptable with both venous and capillary blood samples in field settings.

## Additional file

Additional file 1: Annex 1. Medline Search Strategy - Descriptions of search terms used for literature search in Medline. (DOCX 16 kb)

## Abbreviations

ART: Antiretroviral therapy
DTA: Diagnostic test accuracy
GLLAMM: Generalized latent and linear mixed modeling
HSROC: Hierarchical summary receiver operating characteristic
LMICs: Low and middle income countries
LoA: Limit of agreement
POC: Point-of-care
WHO: The World Health Organization
